# 

**Published:** 1905-09-15

**Authors:** 


### ANNOUNCEMENTS.

First Annual clinic of the Fraternal Dental Society of St. Louis, November 20th and 21st, 1905, at the Barnes Dental College. Special features of this meeting will be a series of lectures on "Cavity Preparation," "Methods and Principles of Packing Gold" and "Methods and Principles of Finishing Fillings," by Dr. E. K. Wedelsteadt, of St. Paul; Drs. A. C. Searl, Owatoma, Minnesota; J. F. Wallace, Canton Missouri, and numerous other members of the Black and Wedelsteadt Clubs and other prominent men in Operative and Prosthetic Dentistry will give clinics. Complete program will be announced later.

All ethical practitioners are invited to be present at clinic. Please send your name and subject of clinic to the Secretary. Exhibit space to be obtained by application to the Secretary.

A cordial invitation is extended to the profession to be present and make this meeting limited in scope but limitless in importance, the best held in this section.

Burton Lee Thorpe, *Pres.*

S. H. Voyles, *Sec'y.*

---

New York Institute of Stomatology Prizes.—With the desire of stimulating investigation in any field of activity directly relating to dental or oral science, the New York Institute of Stomatology offers two prizes for the best papers submitted to it, embodying the results of such original research. The first prize for the best paper will be a gold medal and $250. The second prize for the next best paper, a gold medal and $100.

CONDITIONS.

*a.* The papers offered for competition must be typewritten in English.

*b.* Must contain not less than 1,500 nor more than 3,500 words.

*c.* Must be signed by a motto or *nom de plume.*

*d.* Must be accompanied by a sealed envelope marked with the same motto or nom de plume on the outside, containing the true name as well as the motto of the contestant within.

*e.* Must be sent to the chairman of the Executive Committee, Dr. F. Milton Smith, 38 West 36th Street, on or before March 1, 1906.

JUDGES.

The following gentlemen have consented to act as judges: Dr. C. N. Johnson, of Chicago, Editor of *Dental Review*; Dr. Eugene H. Smith, of Boston, Dean of Harvard University Dental School; Dr. Wilbur F. Litch, of Philadelphia, Editor of *Dental Brief.* Under the following

RULES

1. The papers will be sent to the judges without the sealed envelopes, containing the names of the contestants, which will be retained by the Executive Committee until the decision of the judges is made.

2. In deciding on the merits of papers offered in competition the judges will be requested to take into consideration the value and character of the research work the results of which are represented, more than the literary character of the essays, but to give the latter due credit.

3. The judges are expressly authorized to decide which if any of the papers submitted to them are of sufficient merit to entitle them to the prizes offered, or to withhold the award from all the papers if none are deemed worthy.

4. Authors of the prize papers will be invited to read their essays before a meeting of the institute, as will the writers of other papers of especial merit, the institute reserving the right to the first publication of all papers offered in competition.

Papers not used will be promptly returned to the writers. Those read before the institute will be as fully discussed as possible and when published will be adequately illustrated.

For further information, address Dr. F. Milton Smith, 38 West 36th Street, New York, N. Y.

---

THE AMERICAN SOCIETY OF ORTHODONTISTS will hold its annual meeting at Chicago, September 28th to 30th. No one who is interested in this branch of practice should fail to attend this meeting as it represents the most earnest workers in this specialty.

---

THE NORTHERN INDIANA DENTAL SOCIETY will hold a two-day's session September 19th and 20th, at Logansport.

---

TRANSACTIONS FOURTH INTERNATIONAL DENTAL CONGRESS.—The first volume of these transactions has just been issued in book form by the S. S. White Dental Manufacturing Company. The volume contains names of the officers, list of members by States and Countries, proceedings of the four general sessions and the papers and discussions of sections I and II. The work is well printed, on good paper and bound in paper covers. The other volumes will be issued as fast as practicable. The complete transactions will make a most valuable contribution to our literature.

